# Studying KcsA Channel Clustering Using Single Channel Voltage-Clamp Fluorescence Imaging*

**DOI:** 10.3389/fphys.2022.863375

**Published:** 2022-06-03

**Authors:** Hugo McGuire, Rikard Blunck

**Affiliations:** ^1^ Department of Physics, Université de Montréal, Montréal, QC, Canada; ^2^ Department of Pharmacology and Physiology, Université de Montréal, Montréal, QC, Canada; ^3^ Interdisciplinary Research Center on Brain and Learning (CIRCA), Université de Montréal, Montréal, QC, Canada

**Keywords:** planar lipid bilayer, lateral diffusion, ion channel clustering, membrane curvature, single molecule fluorescence

## Abstract

Oligomerization and complex formation play a key role for many membrane proteins and has been described to influence ion channel function in both neurons and the heart. In this study, we observed clustering of single KcsA channels in planar lipid bilayer using single molecule fluorescence, while simultaneously measuring single channel currents. Clustering coincided with cooperative opening of KcsA. We demonstrate that clustering was not caused by direct protein-protein interactions or hydrophobic mismatch with the lipid environment, as suggested earlier, but was mediated *via* microdomains induced by the channel in the lipid matrix. We found that single channel activity of KcsA requires conically-shaped lipids in the lamellar liquid-crystalline (L_α_) phase, and the need for a negative spontaneous curvature seem to lead to the deformations in the membrane that cause the clustering. The method introduced here will be applicable to follow oligomerization of a wide range of membrane proteins.

## Introduction

Oligomerization is a key event in the physiological function of a number of membrane proteins including signal transduction receptors (Weiss and Littman, 1994; Han et al., 2009; Kawashima et al., 2010; Swift et al., 2011; Sergeev et al., 2012) or pore forming toxins (Collier, 2009; Groulx et al., 2011; Thompson et al., 2011). Similarly, complex formation is a prerequisite for various signaling and transport events triggered by signaling complexes and secretion systems (Cascales and Christie, 2003; Cebecauer et al., 2010). Ion channels, in contrast, are not generally thought to require oligomerization or “clustering” after their assembly in the golgi/ER to function correctly, but some are known to recruit auxiliary subunits. Nevertheless, clustering has been suggested to modify the function of several ion channels or pore forming toxins (Iwasa et al., 1986; Kim et al., 1995; Kaulin et al., 1998; Tiffany et al., 2000; Goforth et al., 2003; Grage et al., 2011; Thompson et al., 2011; Dixon et al., 2012; Olesen et al., 2013; Dixon et al., 2015), including the prokaryotic proton-gated ion channel KcsA (Molina et al., 2006; Sumino et al., 2014). The altered behavior of ion channels in clusters might very well explain inconsistencies when reconciling molecular properties of ion channels with models of the electrical properties of membranes *in situ*.

Oligomerization has been studied previously using different techniques including Förster resonance energy transfer (FRET) and single molecule fluorescence (Kawashima et al., 2010; Groulx et al., 2011; Kasai et al., 2011; Swift et al., 2011; Thompson et al., 2011). However, in the previous studies either the steady state was observed (Groulx et al., 2011) or oligomerization occurred too rapidly to resolve (Thompson et al., 2011). In the present study, we went one step further and directly observed the dynamics of the oligomerization process on the single molecule level and correlated it with functional data. To this end, we performed single molecule voltage-clamp fluorometry imaging, where we follow the diffusion and oligomerization of single fluorescently-labeled KcsA channels while simultaneously recording the function via electrophysiology.

We chose to work in a planar lipid bilayer system as, here, we gain control over the lipid composition of the surrounding membrane and can determine the influence of lipid composition on both ion channel function and clustering. Previous studies suggested modulation of channel activity as a function of several physical properties of the bilayer such as its fluidity, free volume, stiffness and the intrinsic lipid curvature (McIntosh and Simon, 2006; Andersen and Koeppe, 2007). Notably, another important modulator for channel activity is the electrostatic charge of the surrounding lipids. One well studied example of ion channel regulation by charged lipids is the highly-anionic phosphatidylinositols that gate inward rectifying potassium (K_IR_) and ATP-dependent potassium channels (K_ATP_) (Hilgemann and Ball, 1996; McLaughlin et al., 2002; Fürst et al., 2014). The proton-gated KcsA potassium channel has been suggested to require anionic lipids for opening (Heginbotham et al., 1998; Valiyaveetil et al., 2002), whereas voltage-gated potassium channels decrease and increase their open probability in the presence of anionic and cationic lipids, respectively (Schmidt et al., 2006; Börjesson et al., 2008; Faure et al., 2012; Faure et al., 2014).

Also the hydrophobic thickness of the lipid bilayer has been suggested to affect the function of ion channels (Baldwin and Hubbell, 1985; Perozo et al., 2002; Williamson et al., 2002; Yuan et al., 2004; Yuan et al., 2007; Tong et al., 2012; Rusinova et al., 2014). Changes in the thickness alter both the conductance and open probability of BK channels (Yuan et al., 2004; Yuan et al., 2007), as well as the water permeability of aquaporin-4 channels, the primary water channel in the mammalian brain (Tong et al., 2012). Similarly, the energy required to activate the mechanosensitive MscL channels was lowered when reducing bilayer thickness (Perozo et al., 2002). KcsA was suggested to increase its open probability with decreasing chain length (Rusinova et al., 2014).

Since the lipid-mediated effects discussed above possibly co-exist and modulate ion channel function there is an increasing interest in measuring channel activity under simultaneous control of several possible mediators. In this study, we investigated the effect of both bilayer thickness and lipid headgroup properties on KcsA behavior and oligomerization, by simultaneously tracking single channel function and position.

## Materials and Methods

### KcsA Purification, Labeling and Reconstitution in Lipid Vesicles

If not stated otherwise, chemicals were obtained from Sigma-Aldrich. We used the KcsA mutant E71A since this mutation removes both the inactivation occurring at the selectivity filter (C-type inactivation) and the slight voltage dependence of KcsA (Cordero-Morales et al., 2006a; Cordero-Morales et al., 2006b). KcsA was expressed and purified from *Escherichia coli* strain M15 transformed with a N-terminally (His6)-tagged KcsA-E71A-H124C-pQE32 vector as described previously (Blunck et al., 2008), with slight modifications. Cells were first grown overnight in 10 ml LB media supplemented with 100 μg/ml Ampicillin and 25 μg/ml Kanamycin. This preparation was then added to 1L LB media supplemented with 100 μg/ml Ampicillin and grown for about 2.5 h until OD_600_∼0.6. For induction of KcsA expression, the culture was supplemented with 5% glycerol and 5 mM isopropyl β-D-1-thiogalactopyranoside (IPTG) and kept at 25°C for about 4 h. Cells were harvested and homogenized on ice in a previously cooled sodium-phosphate based buffer (NaP_KCl, NaCl_: 100 mm NaCl, 50 mm KCl, 50 mm phosphate buffer containing about 45 mm Na_2_HPO_4_ and 5 mm NaH_2_PO_4_, pH 7.5) supplemented with 1 mm phenylmethylsulfonyl fluoride (PMSF) prior to lysis using an EmulsiFlex-C5. Membranes were isolated by ultracentrifugation at 150,000 g for 1 h and then solubilized in 2% n-Dodecyl β-D-maltoside (DDM) for 2 h at 4°C. The sample was centrifuged at 100,000 g for 30 min to remove unsolubilized fractions.

Purification of the channel was accomplished by Cobalt affinity chromatography (Talon superflow, Clontech) on an ÄKTA purifier (GE Healthcare, life sciences) using 0.02% DDM and 40 mm imidazole in NaP_KCl-NaCl_ buffer supplemented with 5% glycerol. Prior to elution at 400 mM imidazole, the cysteines were reduced by continuous flow (∼1.5 ml/min) of 1 mm Tris (2-carboxyethyl)phosphine hydrochloride (TCEP-HCl, Pierce) for 10 column volumes. The channels were labeled overnight at 4°C with >5x molar excess of either AlexaFluor-488-C_5_-maleimide, AlexaFluor-594-C_5_-maleimide or AlexaFluor-647-C_2_-maleimide. Unreacted dyes were removed by a second purification on the cobalt affinity column.

The channels were reconstituted in lipid vesicles of either DPhPC, POPE:POPG (3:1), di (14:1)-PC, di (16:1)-PC, di (18:1)-PC, di (18:1)-PC: di (18:1)-PG (3:1), di (20:1)-PC, or di (22:1)-PC (Avanti Polar Lipids). All phospholipid mixture were solubilized in chloroform, dried and resuspended in High-K buffer (450mM KCl, 10 mM Hepes, pH 7.4) at 10 mg/ml. Lipid-containing solutions were sonicated until transparency, indicating formation of small unilamellar vesicles. Proteins were added at a 1:100 protein-to-lipid mass ratio, resulting in a channel concentration of about 1.3 µM. Detergent was removed using a D-Tube Dialyzer Mini (Novagen) with a molecular weight cut-off of 12–14 kDa. Samples were dialyzed for at least 3 days in the High-K buffer, changing the solution every 6–12 h. Subsequently, they were aliquoted, flushed with nitrogen and kept either at 4°C up to 3 months or at −80°C for long term storage.

### Supported Bilayers

Experiments with supported bilayers were accomplished as described previously (Blunck et al., 2008; Groulx et al., 2011). Briefly, glass coverslips (Fisherfinest Premium Cover Glass 25 × 25—1, Fisher Scientific) were cleaned by several 30 min sonication steps at 50°C first in Alconox and then in anhydrous ethanol. Coverslips were rinsed in Milli-Q water between each sonication step. Clean coverslips were then glued to an external chamber as support for the bilayers. The chamber was filled with a solution containing 100 mm KCl, 10 mm Hepes and 1 mm CaCl_2_ (pH 4). Before injecting KcsA proteoliposomes, KcsA reconstituted in vesicles of DPhPC were diluted 167-fold, from 1.3 µM to about 9.8 nM in vesicles of the desired composition at 2.5 mg/ml. 3 µL of this preparation was mixed to a chamber containing 400 µL and allowed to reach the coverslip for about 30 min before washing the excess vesicles. Fluorescence recordings of single channels labeled with AlexaFluor-594-C_5_-maleimide were done using an inverted microscope (Axiovert 200, Zeiss) with a high numerical aperture objective (Plan-Apochromat ×40/N.A. = 1.3, Zeiss), an EMCCD camera (iXon^+^ 860BV, Andor Technology) and a 532 nm laser diode (World Star Tech) exciting the dyes at 500 µW.

Analysis of single subunit counting data and the intensity of each fluorescent spot prior to photobleaching was obtained using *PIF* (McGuire et al., 2012), a software for single-subunit counting analysis.

### Voltage-Clamp Fluorescence Imaging Recordings of Single KcsA Channels

**FIGURE 1:**
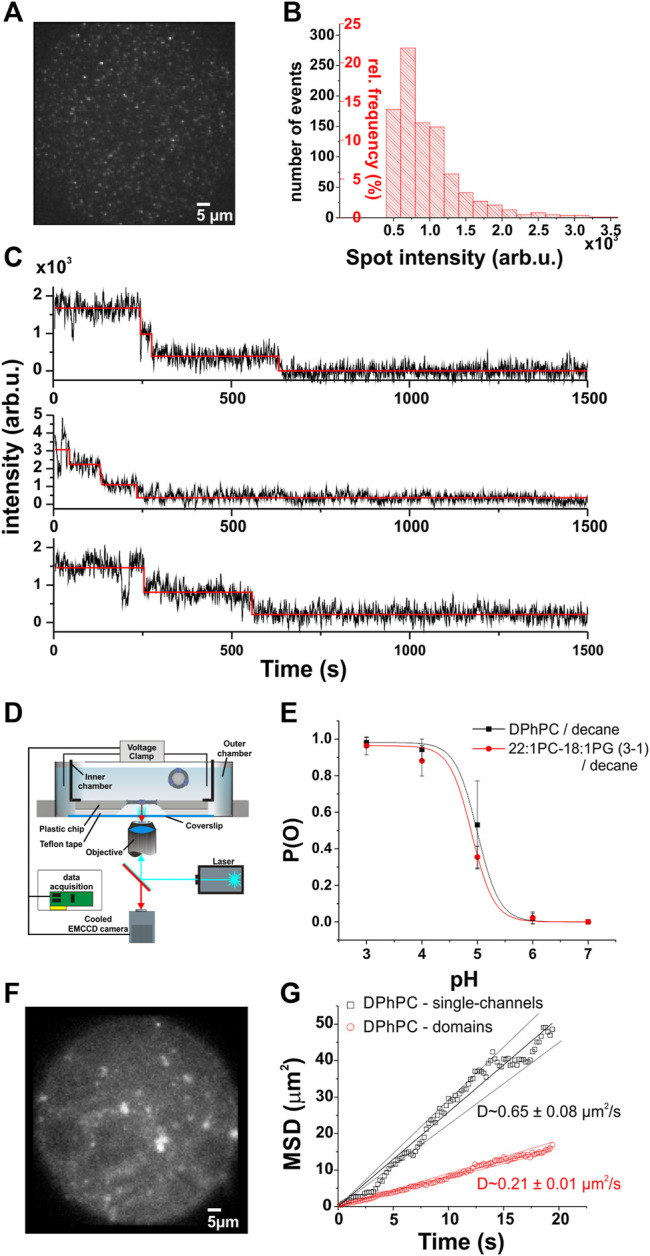
Single-channel voltage-clamp fluorescence imaging. **(A)**, Single KcsA-E71A-H124C channels in DPhPC supported bilayers. **(B)**, The normalized frequency distribution of spot intensities shows a single-channel fluorescence peak ranging from ∼500–3,500 arb.u. Spots were selected only if they showed 1–4 photobleaching steps, as detected by automated analysis software (McGuire et al., 2012). **(C)**, Examples of photobleaching traces from **(A)**. Photobleaching experiments were performed at higher light intensity, and stepwise bleaching analyzed with the PIF software. By analyzing the number of photobleaching steps of each spot found in the supported bilayers, we were able to calibrate the fluorescence intensities to the number of channels. **(D)** *.* The planar lipid bilayer is formed within an aperture of a thin plastic chip (150–250 µM). Solutions are allowed to flow on both sides of the membrane with a teflon spacer between chip and coverslip. To minimize the distance between the objective and the bilayer, a coverlip of only 80 µM thick is separating the outer chamber from the optical system. This configuration permits the use of a high numerical objective (>1.4) together with the use of a standard voltage-clamp setup. Moreover, the channels are completely free of surface interactions, in contrast to supported bilayers where the channels are in close proximity to the supporting surface. The fluorescence and ionic current are simultaneously measured as described in the text. **(E)** *.* Open probability of KcsA-E71A-H124C as a function of pH for two different lipid compositions. Single-channel recordings did not reveal significant changes in the pH dependence of the open probability between DPhPC/decane and 22:1 PC-18:1 PG/decane bilayers (N > 3 for each condition). **(F)**, Example of KcsA channels in planar lipid bilayer (DPhPC/decane). **(G)**, The mean squared displacement (MSD) as a function of time to illustrate the diffusion of KcsA channels in planar lipid bilayer. The analysis was separated in two groups, since low intensity single-spots, constituting single-channels (intensity < 3500 AU), appear to diffuse faster than larger fluorescent domains as expected for Brownian diffusion. The average diffusion constant evaluated from the slopes is 0.65 ± 0.08 µm^2^/s for single-channels (*n* = 16) and 0.21 ± 0.01 µm^2^/s for larger domains (*n* = 132). The errors were estimated according to the confidence levels shown as dashed lines.

Voltage-clamp recording was combined with a fluorescence microscope described above to allow simultaneous observation of current and fluorescence of the KcsA channels (Figure 1D). To allow optical access to the lipid bilayer, a cylindrical shaped chamber (outer chamber) carrying a round glass coverslip (thickness #0: 0.08 mm; VWR international) over its bottom side was placed on top the sample holder. A smaller cylindrical chamber (inner chamber) holding a < 80 µM thick acetal film (McMaster-Carr) underneath was placed inside the first chamber. The film contained a 150–250 µM-diameter aperture, in which the bilayer was formed. Solution containing 100 mm KCl, 10 mm Hepes and 1 mm CaCl_2_ (pH 4) was added to both chambers before the formation of the lipid bilayer in the hole. Prior to all lipid bilayer experiments, a drop of decane-solubilized lipids was placed on the rim of the aperture and dried exposed to air. The planar lipid bilayer was formed according to the “painted bilayer technique” by applying either decane or hexadecane solubilized phospholipids at a concentration of 25 mg/ml over the hole using a glass rod. A membrane potential of 100 mV was applied in order to detect ion conduction. Channels were added to the lipid bilayer by injecting about 0.15–0.25 μL of KcsA reconstituted in lipid vesicles (0.1 mg/ml) in the inner chamber. Once channel activity was detected, excess vesicles were removed from the inner chamber by washing 5–10 times. The system was connected to an Axopatch 200A amplifier and externally controlled using GPatchM software. The time-resolved fluorescence imaging was acquired simultaneously by the EMCCD camera triggered from GPatchM. The labeled channels were fluorescently excited by a 500 µW-laser beam with a wavelength of either 488 nm, 532 nm or 635 nm. Emitted light was filtered by a bandpass filter (525/50, 605/70 or 705/80, Chroma Technologies) combined with a dichroic mirror (405/488/561/635 nm lasers BrightLine quad-edge laser-flat, Semrock, or z532, Chroma Technologies). Exposure time for recordings was set to 200 ms. Change of pH on a single side of the bilayer confirmed a preferred orientation (>95%) of the KcsA channels in the bilayer.

Tracking of single-channel diffusing in a lipid bilayer—The channels incorporated in the lipid bilayer were freely laterally diffusing. Single bright spots corresponding to either clustered channels or single-channels could be tracked using an in-house program written in Matlab (Mathworks). The lateral diffusion coefficient *D* was calculated from the slope (*D* = slope/4) of the mean square displacement $\langle r^{2}\rangle$ as a function of time *t* using channel trajectories:

$$\langle r^{2}\rangle =4Dt$$ (1)

The intensity value of each spot was tracked over time and was used to evaluate the presence and number of single or clustered channels. The background of a spot was removed at each frame by fitting the spot to a 2-D Gaussian and subtracting the calculated baseline.

Determining FRET efficiency and evaluating distances between single FRET pairs—To determine the FRET efficiency *E* and the corresponding distance *r* between a donor and an acceptor, we first evaluated the FRET intensity by exciting the donor and measuring the intensity of the acceptor. Here, we used the AlexaFluor-488-C_5_-maleimide for the donor and AlexaFluor-647-C_2_-maleimide for the acceptor. A leak of the donor emission could be directly detected when measuring FRET using the acceptor bandpass filter. Also, the acceptor could be slightly excited by the light used to excite the donor, resulting in a leak of the acceptor emission. We therefore corrected for these possible situations using the following equation.

$$FRET=I_{488-705/80}^{DA}-I_{488-525/50}^{DA}\cdot leakDonor-I_{635-705/80}^{DA}\cdot leakAcceptor$$ (2)

$I_{y-z}^{x}$ is the intensity of *x* while exciting at *y* nm after filtering with the *z* filter. *x* could be the donor alone (*D*), the acceptor alone (*A*), or the donor-acceptor pair (*DA*). *y* is either 488 or 635, depending if exciting the donor or the acceptor, respectively. *z* represents the filter used. For the donor or acceptor fluorescence, the bandpass filter 525/50 or 705/80 was used, respectively, in combination with the multi-edge dichroic beamsplitter (405/488/561/635). *leakDonor* and *leakAcceptor* were both evaluated before the FRET experiments using either the donor or the acceptor alone. They can be represented by the following equations.

$$leakDonor=\frac{I_{488-705/80}^{D}}{I_{488-525/50}^{D}}$$ (3)

$$leakAcceptor=\frac{I_{488-705/80}^{A}}{I_{635-705/80}^{A}}$$ (4)

In respect of our experimental conditions and using constant laser intensities, we found *leakDonor = 0.011 ± 0.003* and *leakAcceptor = 0.08 ± 0.02* (mean ± stddev). From equations (2), (3) and (4) *E* and *r* can be evaluated by using these equations.

$$E=\frac{FRET/\left(QY_{A}\cdot CorrFiltA\right)}{FRET/\left(QY_{A}\cdot CorrFiltA\right)+I_{488-525/50M}^{DA}/\left(QY_{D}\cdot CorrFiltD\right)}$$ (5)

r=R0(1−EE)16 (6)

Here, *QY*_*D*_ *= 0.92* and *QY*_*A*_ *= 0.33* are the quantum yields of the donor and the acceptor, respectively. Since only a fraction of either the donor or the acceptor spectra was collected, a correction factor for the filters was added (*CorrFiltD = 0.66* and *CorrFiltA = 0.49*) *R*_*0*_ *= 56 Å* is the distance between the FRET pair for which the FRET efficiency is *E = 50%*.

When two channels separated by a distance *r* are fully labeled, one with the donor one other with the acceptor, the apparent distance *r* as seen in FRET appears generally higher. The pore size was estimated to 46 Å and the distance between labels to 18 Å (Figure 3C, *left*). The relationship between the real and apparent distance *r* is shown in Figure 3C (*right*). An energy transfer rate is calculated as $k_{ij}=\frac{1}{\tau_{D}}{\left(\frac{R_{0}}{d_{ij}}\right)}^{6}$ based on the separating distance *d* for each donor (*i)*-acceptor (*j)* combination. For each donor, the transfer efficiency can be evaluated by Ei=∑jkij∑jkij+1τD . Since every donor undergoes the same process, the apparent transfer efficiency becomes $E=\frac{\sum_{i}^{N}E_{i}}{N}$ for a total of *N* donors. The corresponding apparent distance *r* is then calculated as r=R0(1−EE)16 .

## Results

### Calibration of Single Channel Fluorescence Intensities

Before tracking single channel oligomerization, we had to establish what constitutes a single channel and multiple channels in terms of fluorescence intensity. We therefore calibrated the fluorescence intensity originating from a single channel in DPhPC (1,2-diphytanoyl-sn-glycero-3-phosphocholine) supported lipid bilayer as described previously (Blunck et al., 2008) (Figures 1A–C). The advantage of supported lipid bilayers is that channels are prevented from diffusing in the bilayer and thus from clustering. We attached a thiol-reactive fluorescent probe at position H124C in the KcsA-E71A background and determined the distribution of spot intensities for the entire field of view at the observation excitation intensity of 500 µW. We then increased the intensity to 850 µW in order to induce faster and complete photobleaching and determined for each spot the number of fluorophores using single subunit counting (McGuire et al., 2012; Blunck, 2021) (Figure 1C). Spots were classified as single channels if their fluorescence decayed in 1-4 bleaching steps in accordance with the tetrameric structure of the KcsA channels. Each of the four monomers is labeled with a single fluorophore. Intensity values in the range between 500–3,500 arb.u. were found to be characteristic for a single channel (Figure 1B).

### Channel Diffusion and Direct Observation of Single-Channel Clustering in Planar Lipid Bilayers

With this calibration, we next aimed to track the single channels in a lipid membrane where they are free to diffuse. We formed planar lipid bilayers according to the painted bilayer method (Mueller et al., 1962). The bilayers were formed in a small aperture in polymer chips that place the bilayer within the working distance of a high numerical aperture objective (Ries et al., 2004; Groulx et al., 2010). We tracked the single channels by imaging the fluorescence of the bilayer with an EMCCD camera while simultaneously recording the electrical activity (Figure 1D). In order to keep the channel in a mainly activated-open state, the proton concentration on both sides of the lipid bilayer was adjusted to pH 4 (Cordero-Morales et al., 2006a; Chakrapani et al., 2007; Thompson et al., 2008; Iwamoto and Oiki, 2013). The high open probability *p*_*O*_ simplified the assessment of channel activity. Channels were fused to bilayers composed of DPhPC or a lipid mixture of 1-palmitoyl-2-oleoyl-sn-glycero-3-phosphoethanolamine and 1-palmitoyl-2-oleoyl-sn-glycero-3-phospho- (1′-rac-glycerol), POPE: POPG (3:1 w:w) in decane. The mutation H124C with the linked dye did not affect normal KcsA activity at pH 4 (Figure 1E). The open probability of the channel was close to one in both lipid compositions as expected for a functional KcsA-E71A at pH 4.

**FIGURE 2:**
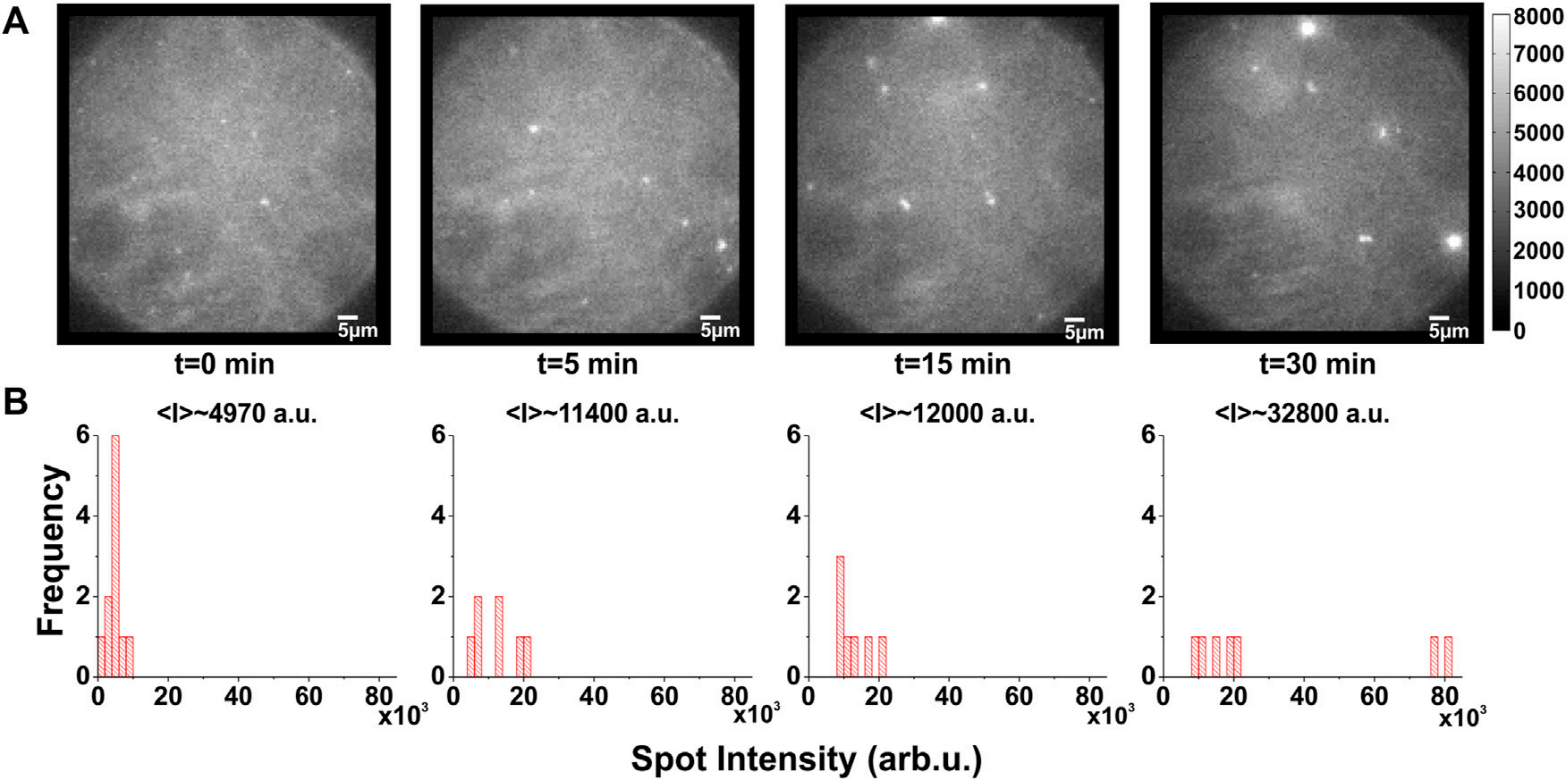
Temporal evolution of KcsA clustering. **(A,B)**, Example of typical time course of clustering: **(A)**, images taken from a DPhPC/decane bilayer in which labeled KcsA channels were fused show that clustering appears more and more prominent over time. **(B)**, the corresponding spot intensity distribution confirms a shift towards clustered spots, although some could remain individual. Excitation light was restricted to the central area of the bilayer in order to prevent scattering from the torus and the chamber. This and inevitable photobleaching meant that the total number of channels is not necessarily constant.

In the fluorescence signal, we observed, in addition to the single spots, highly fluorescent “spots” that contained a larger number of channels (Figure 1F). These highly fluorescent areas increased in number and intensity over time (Figure 2A), suggesting that they represent clusters of KcsA channels that slowly agglomerate. We quantified this by determining the temporal development of the spot intensity of the bilayers. When plotting the spot intensity distribution of a single bilayer over time, one can follow the agglomeration of channels in fewer but more intense spots (Figure 2A). After about 10–30 min, most channels assembled in clusters. Accordingly, the average intensity of the spots had increased 6-7-fold from 4,970 a.u. (1–3 channels) to 32,800 a.u. (10–20 channels; Figure 2B). While the exact number of spots and clusters varied between different bilayers, the clustering always occurred in this manner.

### Tracking Diffusion and Clustering of Single KcsA Channels

By directly tracking the position of single spots, we were also able to characterize the diffusion behavior of single channels and higher oligomers (Figure 1G). In DPhPC, lateral diffusion coefficients of 0.65 ± 0.08 and 0.21 ± 0.01 µm^2^/s were obtained for single channels and clusters, respectively. The clusters diffused significantly slower than the single channels due to their larger radius. A 3-fold lower diffusion coefficient would be consistent with 7–9 channels per cluster in average. However, the clusters were not of uniform size and the diffusion coefficient thus represents an average.

**FIGURE 3:**
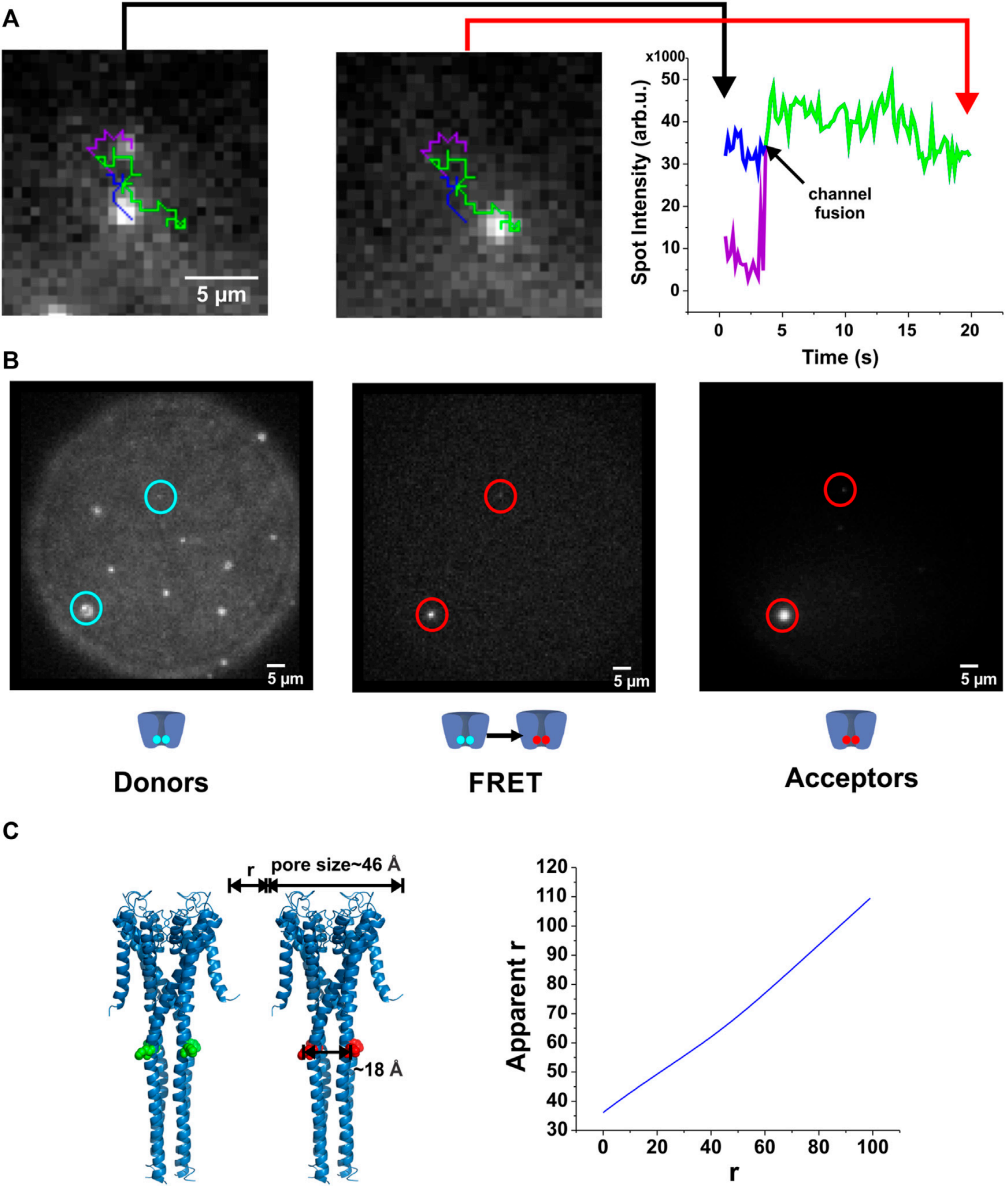
Direct observation of KcsA clustering. **(A)**, By tracking the fluorescence of individual spots, their interaction has been assessed. When two channels fuse, the channels remain in close contact and the total intensity corresponds to the sum of the individual spots. **(B)**, *FRET occurs between co-localized channels.* KcsA channels were labeled either with AlexaFluor-488-C_5_-maleimide (donor) or AlexaFluor-647-C_2_-maleimide (acceptor). As the R_0_ of this fluorescent pair is about 56 Å, we were able to evaluate the proximity from about 35 Å to 85 Å. Both populations were injected one after the other on the planar lipid bilayer. After a few minutes, co-localized donor-acceptor pair was observed (*left and right panels*), as well as a low FRET signal (*middle panel*), indicating channel proximity (corrected r) of about 60 Å (*n* = 33). Our results suggest therefore that direct protein-protein interactions do not occur, although they assemble in closely packed microdomains. **(C)** *.* Illustration of the influence of multi-labeling when evaluating distances by FRET measurements (see methods for details).

If the single channels clustered over a period of 30 min, we should be able to directly observe fusion between two single channels. We therefore tracked the diffusion of single channels proximal to one another until fusion was observed (Figure 3A; Supplementary Movie S1). After fusion, the fluorescence intensity of the “dimer” spot equaled the sum of the intensities of the two original spots, indicating that indeed both proteins diffused collectively in the newly formed cluster (Figure 3A).

### KcsA Clusters With Significant Distance Separating the Channels

Channel clusters could be stabilized either by directly interacting (protein-protein interaction) as suggested previously (Kim et al., 1995; Tiffany et al., 2000; Olesen et al., 2013; Visscher et al., 2017), or they could interact *via* the membrane environment. From our observations, we can deduce that the two channels enter within the radius of a diffraction limited spot (∼300 nm) preventing us from optically distinguishing two spots. To estimate a lower limit for the distance between two channels, we performed Förster Resonance Energy Transfer (FRET) measurements. We simultaneously fused DPhPC liposomes containing channels labeled with either AlexaFluor-488-C_5_-maleimide or AlexaFluor-647-C_2_-maleimide (R_0_ = 56 Å) to the lipid bilayer and performed FRET measurements by exciting the donor (at 488 nm) and imaging the acceptor emission (705 nm/40 nm^1^; sensitized emission).

Soon after vesicle fusion to the lipid bilayer, spots containing both donor- and acceptor-labeled channels were observed and gave rise to sensitized emission. Over time, with increasing number of channels, more and more spots developed a sensitized emission signal (Figure 3B). The mean energy transfer efficiency was *E* = 0.11 ± 0.08 (mean ± stddev). Such an energy transfer corresponds to a distance between two channels of approximately *r* ∼ 60 Å. This calculates the apparent energy transfer between two KcsA channels with a closest distance *r* and fully labeled at position H124C (for details see Figure 3C). This approximation only considers two channels, as a higher number of channels would result in too many geometric combinations. However, we did not observe higher energy transfer for those spots containing just two channels compared to the larger spots, indicating that the multiple geometric arrangements had no significant influence on energy transfer efficiency. We also analyzed the energy transfer efficiency for each single spot so that unpaired channels or clusters containing only donors or only acceptors did not influence the transfer efficiency. The relatively large distance of 60 Å (chain of ∼15 lipid molecules) between neighboring channels suggests that no direct protein-protein interaction. Combined with the optical resolution limit the distance between channels in a cluster is at least 60 Å and less than 300 nm.

### Channel Clustering Occurs for Various Bilayer Thickness Values

Both the apparent distance between two clustered KcsA channels and the “long-range interaction” allowing oligomerization indicate that clustering is promoted by an interaction with the environment and not a direct protein-protein interaction. One possible explanation would be a local deformation in terms of curvature or thickness or phase in a microenvironment around the channel. This change in environment would be linked to an energy cost, which would be reduced if shared by several channels, generating the driving force for the clustering.

A mismatch in the hydrophobic surface of the channels and the hydrophobic thickness of the bilayer could lead to a compression or expansion of the bilayer (Dan et al., 1994; Kralchevsky et al., 1995; Mouritsen and Bloom, 1984; Botelho et al., 2006; Lewis and Engelman, 1983a; Sparr et al., 2005). We have two possible values for the hydrophobic thickness native to KcsA. According to the full length crystal structures (Uysal et al., 2011; Uysal et al., 2009) the hydrophobic thickness^2^ would be 34 Å and 33 Å for the open and closed state, respectively. However, the membrane of *Streptomyces lividans*, the actinobacteria from which KcsA was originally obtained, contains mainly branched-chain saturated C14, C15 and C16 iso-acids and C15 anteiso-acids (Verma and Khuller, 1983). Their thickness should be close to DPhPCs, which is also a C16 lipid with branched hydrocarbon chains. DPhPC’s hydrophobic thickness at T = 20°C is 27.8 Å (Kucerka et al., 2011). We thus estimate the native hydrophobic thickness for KcsA 28–34 Å.

We varied the thickness of PC bilayers by 1) altering the acyl chain length from 14:1 to 22:1 and 2) using decane and hexadecane as solvent for the painted bilayer. In solvent-free bilayers, the hydrophobic thickness can be estimated as (n − 1)1.75 **Å**, where *n* is the number of carbons in the acyl chain (Lewis and Engelman, 1983b). The solvent adds to this thickness due to residual solvent in the bilayers. Hexadecane adds ∼10% to the bilayer thickness, whereas decane adds more. In a painted lipid bilayer, the experimental thickness of the DPhPC/decane bilayer was previously estimated to be 44 Å (Gross et al., 2011). We tested conditions from 25 to 58 Å (Table 1) (Gross et al., 2011; Benz et al., 1975). As the lipids of reconstitution might have a significant effect on channel behaviour (Faure et al., 2014), we reconstituted the channels directly in lipids of the same composition as the bilayer during the experiments.

**TABLE 1:** Estimated thicknesses of the bilayer/solvent combinations tested in the clustering experiments. The two shortest chain lengths (14:1 and 16:1) did not produce stable bilayers, so no results have been obtained. For all other conditions, clustering was observed.

Chain length	Solvent	Estimated Thickness (Å)	Clustering
14:1	hexadecane	25	-
	decane	38	yes
16:1	hexadecane	29	-
	decane	42	yes
18:1	hexadecane	33	yes
	decane	48	yes
20:1	hexadecane	36	yes
	decane	54	yes
22:1	hexadecane	40	yes
	decane	58	yes
DPhPC	hexadecane	31	yes
	decane	44	yes

**FIGURE 4:**
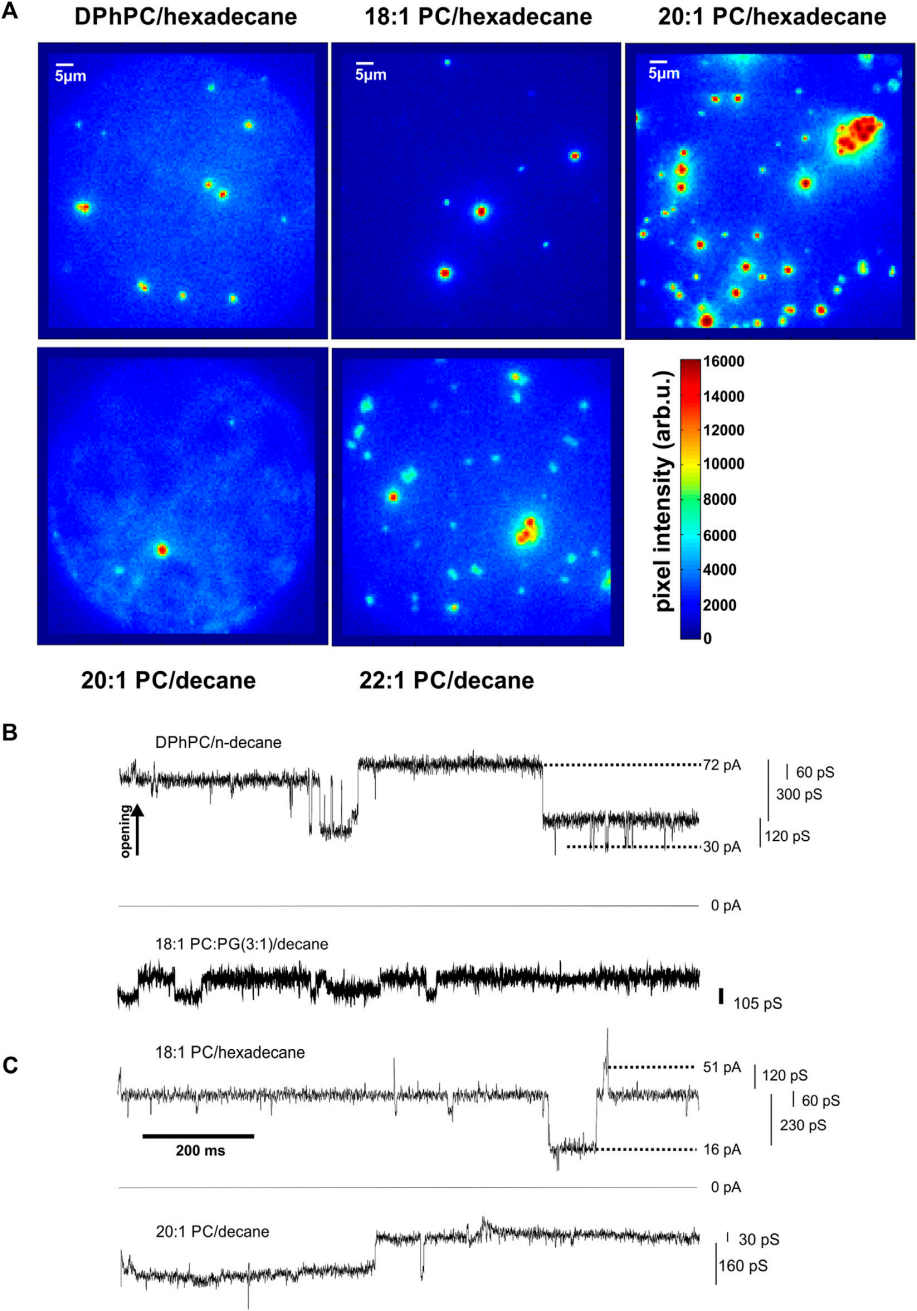
KcsA clustering is observed for various thickness conditions. **(A)**, Images taken from *top* DPhPC/hexadecane bilayers (*left*, *n* = 10), 18:1 PC/hexadecane bilayers (*middle*, *n* = 10), 20:1 PC/hexadecane bilayers (*right*, *n* = 4) and *bottom* 20–1/decane (*left*, *n* = 2) and 22–1/decane (*right*, *n* = 6). While changing the solvent between n-decane and hexadecane and varying the acyl chain length of the lipid bilayer, changes in bilayer thickness are expected. Since clustering was observed for all tested conditions, from about 30 Å to about 58 Å (Benz et al., 1975), it appears that hydrophobic mismatch does not occur or does not influence significantly KcsA clustering in our experiments. Differences in number of clusters depend on total number of channels in the bilayers and showed no dependence on lipid/solvent combination. **(B,C)**, *Coupled KcsA gating involving several channels.* Cooperative activity involving several channels was observed in the presence of clusters in bilayers formed of *b* DPhPC/decane and 18:1 PC:PG (3:1)/decane; *c* di (18:1)-PC/hexadecane and 20:1PC/decane (N > 25).

The shortest acyls chains di (14:1)-di (16:1)-PC did not yield stable bilayers in hexadecane, thus the shortest stable bilayers were 30 Å. For all bilayer thicknesses that we tested, we observed the clustering behaviour with similar time courses (Figure 4A). However, time course and number of clusters varied between single experiments and strongly depended on the number of channels initially incorporated into the bilayer. At very high channel concentrations, clusters started to adhere to each other (see for instance Figure 4A, *top right¸*20:1-PC/hexadecane).

The fact that clustering was observed under all experimental conditions (30–58 Å) indicated that hydrophobic mismatch is not the main driving force for clustering. One possible explanation might be that the channel proteins adapt to the different hydrophobic thicknesses rather than compressing or extending the bilayer thickness.

### Relation Between Channel Clustering and Activity

To further narrow down on the reason for clustering, we turned to functional measurements. It had been suggested earlier that clustering influences KcsA function (Molina et al., 2006). In order to characterize the effect of different lipid compositions on ion channel function and its relation to clustering, we imaged channels in the bilayer while simultaneously recording the ionic current passing through it. We first investigated the single channel behaviour in different lipid compositions at low channel density. KcsA-E71A-H124C in a 3:1 (w:w) ratio of di (18:1)-PC:di (18:1)-PG as well as di (22:1)-PC:di (18:1)-PG showed the same high open probability as in DPhPC or POPE:POPG (3:1). There was also no significant difference in the pH dependence of DPhPC and di (22:1)-PC:di (18:1)-PG (Figure 1E) compared to known dependence in POPE/POPG (3:1) (Thompson et al., 2008).

In DPhPC/decane and di (18:1)-PC/PG/decane bilayers (Figure 4B), we observed, in addition to the normal single channel activity, cooperative gating events with conductances multiple to the single channel conductance. However, at the high channel densities that we had to impose in order to observe clustering in a reasonable time frame, it was difficult to distinguish cooperative activity from single channel gating observed simultaneously. We suppressed single channel activity by removing the anionic PG in di (18:1)-PC, making use of the observation that single KcsA channels do not open in pure PC bilayers (Heginbotham et al., 1998; Valiyaveetil et al., 2002). We found that cooperative openings still occurred even in the absence of anionic lipids. In the di (18:1)-PC/hexadecane bilayers [32 Å (Benz et al., 1975)], the channels underwent frequent cooperative openings (Figure 4C). As observed in the presence of PG, the conductance values were multiples of the single channel conductance (5–13 times; Figure 4C). Most importantly, the cooperative openings only occurred if, at the same time, clusters were observed optically. The strong correlation between the occurrence of clusters and cooperative gating suggested that channel opening is coupled in the clusters.

The combination of a high channel density and the limited time that we can observe single channels prevented the direct correlation of the first cooperative gating event with the oligomerization of two channels. We cannot continuously record the channels over a time of 30 min but rather recorded fluorescence in specific time intervals. Once a cooperative event was observed, we no longer can assign the fusion to a cooperative event, as any other fused channel could be responsible. We therefore turned our attention to the relation between KcsA activity and lipid composition.

### KcsA Activity Is Determined by Lipid Phase and Curvature

**FIGURE 5:**
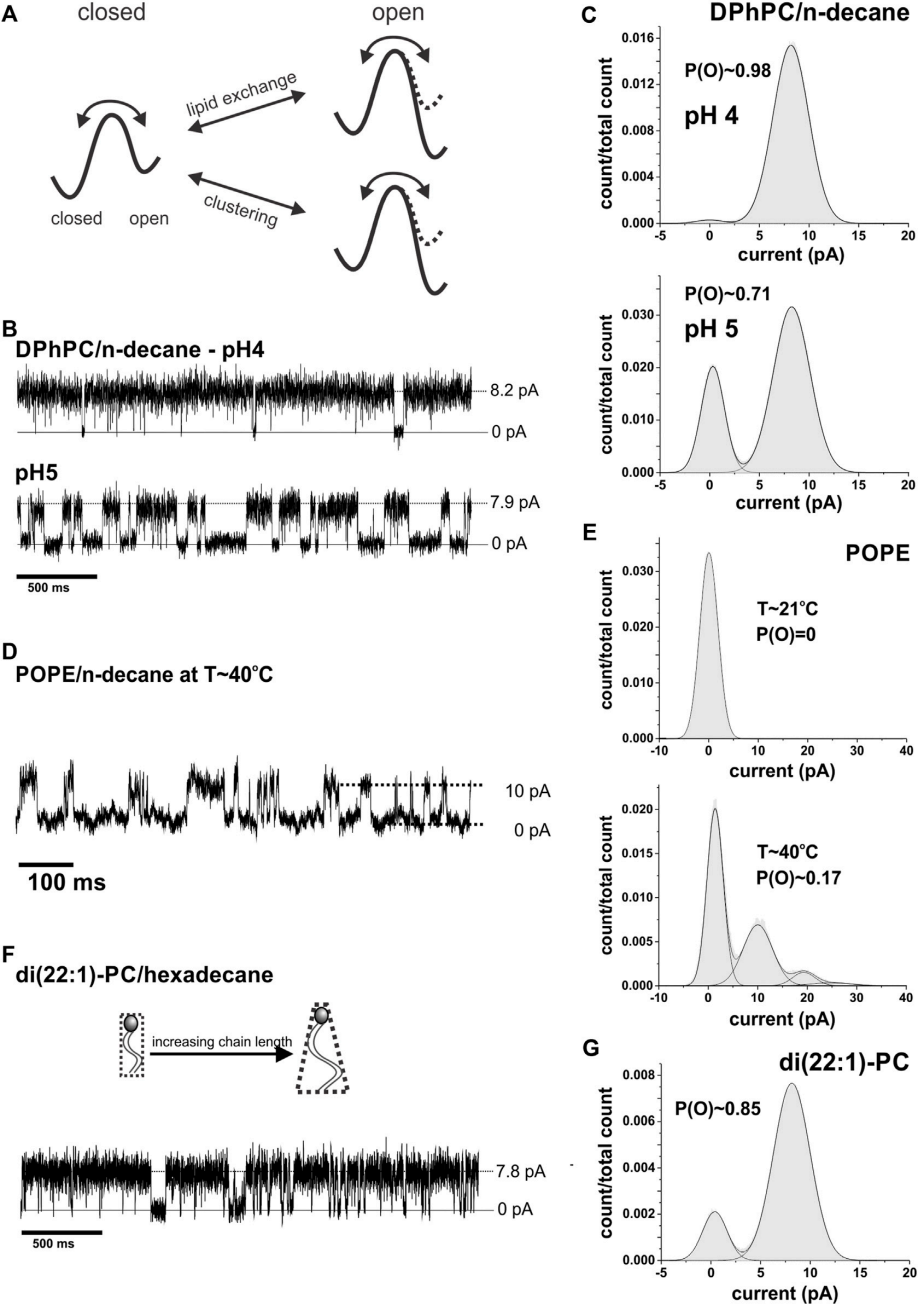
Lipid dependence of single channel activity. **(A)**, Effect of lipid composition and clustering on the energy barrier for KcsA opening. While in a DOPC:DOPG mixture KcsA is active, i.e., the free energy is minimal in the open state, they become inactive in pure DOPC and the free energy is minimal in the closed state. The opening is thus limited by the lipid composition. This limitation is overcome by clustering; clustered channel again have minimal energy for the (cooperative) open state. **(B)**, Representative current traces of single channel activity of KcsA in pure DPhPC bilayers at pH 4 & 5 (N > 25). **(C)**, Amplitude histograms of traces shown in **(B)** *.* The open probability was found to be close to 1 at pH 4. **(D)**, An extract of a current recording of KcsA in pure POPE/decane at high temperature (T∼40°C) is shown. The amplitude histogram is shown in e; in total 5 channels were present in the recording. **(E)**, Current amplitude histograms of KcsA channels in pure POPE at room temperature (T∼21°C, *top*) and after heating up the system to 40°C (*bottom*). The open probability increased from 0 to ∼0.17 with increasing temperature (*n* = 4). **(F)**, Example of a current trace of KcsA in di (22:1)-PC/hexadecane (*n* = 3). **(G)**, Amplitude histogram of the recording shown in **(F)**.

Our above results indicate that cooperative opening of KcsA not only coincides with clustering of the KcsA channels but occurs even in lipid compositions that prevented single KcsA channels from opening such as pure DOPC (di (18:1)-PC) bilayers. This will help us elucidate which parameter is responsible for the clustering because, according to our FRET experiments, also the clustering is mediated via the lipid matrix. Both clustering and lipid composition alter the physicochemical parameters of the lipid matrix to allow channel opening. If we consider opening of KcsA in terms of the free energy of the system, then removing the anionic lipid DOPG from the DOPC/DOPG mixture shifts the equilibrium from the open to the closed state (Figure 5A). Under these conditions, the limiting step for opening is governed by the lipid environment. This limiting step is overcome by clustering, allowing (cooperative) opening of KcsA under identical conditions, i.e., even in DOPC, suggesting that the same physicochemical parameter of the lipid environment which limits KcsA single channel acitivity is also responsible for clustering (Figure 5A). It will therefore be instructive to more closely examine which physicochemical parameters facilitate single KcsA activity.

We already examined the hydrophobic thickness above, and established that bilayers of different thicknesses, within the range tested, neither prevented clustering (Figure 4A) nor opening of single channels (Figure 1E). It is known that single KcsA channels are not active in pure POPE but require POPG to be added in order to gate (Heginbotham et al., 1998; Valiyaveetil et al., 2002). POPG is negatively charged, and it was previously thought that negative charges are essential for KcsA gating (Heginbotham et al., 1998; Valiyaveetil et al., 2002). While the mechanism was uncertain, it was suspected to be related to the local proton concentration or a direct interaction with positively charged residues stabilizing the open state as suggested by Iwamoto and Oiki (Iwamoto and Oiki, 2013). However, we and others (Blunck et al., 2008; Rotem et al., 2010) observed that also in the absence of negatively charged lipids but in the presence of branched acyl chains in DPhPC, the single channels gate normally with an open probability close to 1 (Figures 5B,C). It seems that the branched acyl chains of DPhPC are able to stabilize the open state of KcsA similar to POPG.

We next considered which other parameter of POPE is altered by addition of POPG. POPE has an acyl chain melting temperature T_m_ (lamellar gel L_β_ to lamellar liquid crystalline phase L_α_) of 25°C, which is above the experimental temperature (21°C, Table 2). T_m_ is lowered in the presence of 25% POPG to 20–22°C (Pozo Navas et al., 2005), suggesting that a lipid matrix in the L_α_ phase might be required for channel opening. However, KcsA is inactive in pure POPC (1-palmitoyl-2-oleoyl-sn-glycero-3-phosphocholine) bilayers (Iwamoto and Oiki, 2013) although POPC, similar to POPG, has a chain melting temperature of T_m_=−2°C. So, while the L_β_ phase seems to prevent gating, a lower phase transition temperature alone is not sufficient to allow KcsA gating.

**TABLE 2:** Dependence of single channel activity of KcsA on lipid composition measured in painted lipid bilayers.

Lipid Composition	T (°C)	Phase	Curvature	KcsA Activity
POPE	21	Lβ	conical	no
	40	Lα	conical	yes
POPE:POPG 3:1	21	Lα	conical	yes
DPhPC	21	Lα	conical	yes
POPC	21	Lα	cylindrical	no
22:1-PC	21	Lα	conical	yes

POPG also has a smaller headgroup than POPC and thus a more conical shape. The conical shape favors deformations in the membrane, as they have a higher propensity to form inverted structures (negative spontaneous curvature). Consistently, also DPhPC has a negative spontaneous curvature (Hsieh et al., 1997; Yang and Huang, 2002). Nevertheless, the conical shape of the lipids alone can also not be responsible for the channel activity because no activity is observed in pure POPE bilayers despite the fact that POPE has a strong negative spontaneous curvature. We therefore hypothesize that channel activity of KcsA requires lipids both in the Lα phase and with conical shape. If this hypothesis is true, KcsA should become active if we bring POPE into the Lα phase or if we induce a negative spontaneous curvature in PC bilayers (Table 2).

We tested this hypothesis by incorporating KcsA channels in pure POPE bilayers and increasing the temperature above the chain melting temperature T_m_. Consistent with our hypothesis, KcsA channels became active at temperatures above T_m_ (Figures 5D,E). We further corroborated the need for conically-shaped lipids by testing phosphatidylcholine with a longer chain length since the conical shape of lipids increases with the length of the acyl chains. In di-(22:1)-PC/hexadecane, we found KcsA activity with an open probability *P*(O) ∼ 0.85 (Figures 5F,G). However, at this chain length, fusion seems more difficult, and activity was only observed in 33% of successful bilayer formation. The finding is additionally supported by a previous study showing that cardiolipin, a strong inducer of negative curvature, in a 1:3 mixture with POPE increased KcsA activity ∼3-fold compared to POPG in spite of having the identical charge density (Heginbotham et al., 1998). In summary, these results confirm that lipids in the L_α_ phase and negative spontaneous curvature facilitate KcsA activity.

## Discussion

In this work, we studied the clustering of KcsA by imaging single channels in a planar lipid bilayer while simultaneously recording the ionic current. Our FRET measurements confirmed that the channels uphold a mean distance around 60 Å in the clusters, consistent with measurements using atomic force microscopy (30–140 Å at pH 4) (Sumino et al., 2014). These distances point towards the lipid matrix as the mediator for clustering rather than a direct protein-protein interaction. If the membrane has to be deformed within a microenvironment around the channel, then fusing two of these microdomains together lowers the free energy required by each ion channel. This situation is therefore energetically more favorable.

Previously, deformation of membranes was often suggested to be caused by a mismatch between the hydrophobic thickness of the bilayer and the hydrophobic surface of the proteins. Here, we excluded this possibility, as clustering occurred at all hydrophobic thicknesses that were tested (30–58 Å). Prominent distortion of the membrane thickness were primarily observed for β-barrel proteins such as OmpF (East and Lee, 1982; Williamson et al., 2002; Powl et al., 2005). Immense energy is required to compress or extend the height of a β-barrel membrane protein (=hydrophobic surface), because the secondary structure is stabilized by a high number of hydrogen bonds, most of which would have to be broken simultaneously. This is not the case for α-helical membrane proteins such as KcsA, where the secondary structure remains intact when the helices tilt a bit more. α-helical membrane proteins can thus adapt to changes in membrane thickness as long as the helices are sufficiently long and the structural integrity is not altered. Beyond this range, also α-helical membrane proteins will thin the membrane (Rondelli et al., 2018; Callahan et al., 2019).

In the last years, the membrane curvature has moved into the focus of membrane protein research (Iversen et al., 2015; Tieleman et al., 2021), one prominent example being the exact arrangement of the ATPases in the tubular cristae of mitochondria (Blum et al., 2019) or the mechanosensitive Piezo channels (Guo and MacKinnon, 2017). Also coarse-grained simulations of a voltage-gated ion channel, KvAP, suggest deformation of the surrounding membrane (Tieleman et al., 2021). It has been experimentally shown that the curvature leads to a sorting of KvAP channels according to the preferred curvature (Quemeneur et al., 2014). Unfortunately, we cannot directly control membrane curvature in a planar lipid bilayer system. We will therefore consider how the lipids affect the function of KcsA channels. Rhodopsin photoactivation is, for instance, favored by higher curvature lipids (Soubias et al., 2010). Since lipids influence the ion channel function and, vice versa, the channel induces changes in the surrounding lipid matrix, the energy required for the channel opening or mechanical deformation of the membrane, respectively, will act in both directions such that both processes are likely based on the same physicochemical property; in other words, they are the two outcomes of the same interaction. This is corroborated by the fact that clustering coincided with cooperative gating of KcsA, when the energetic “load” on the channel is reduced.

Our results suggest that single KcsA channels require conically shaped lipids in the lamellar liquid crystal phase to open because more cylindrically shaped lipids (e.g., DOPC) or lipids in the L_β_ phase (e.g., POPE) limit single channel activity. These requirements may be applicable to a wider range of ion channels. For instance, a combination of a negative headgroup charge and a conical shape was suggested to be essential for BK_Ca_ channel gating (Blunck et al., 2001; Crowley et al., 2005).

The clustering is facilitated by the microenvironments around the channels. We can exclude lipid rafts within the membranes, as in most experiments, the membrane was composed of a single type of synthetic lipid. However, there remains the possibility that clusters induced, in addition to the curvature, a phase transition in the immediate lipid environment around themselves.

One possible explanation for the elevated energy cost for opening in the absence of conically shaped lipids would be higher lateral pressure on the helical bundle crossing. In EPR measurements, it has been shown that macromolecules will adopt preferentially that conformation that occupies a smaller volume when under pressure (McCoy and Hubbell, 2011). The helical bundle crossing of KcsA has to widen to open the pore thus pushing against the headgroups of the surrounding lipids. In conically-shaped lipids, the lateral pressure on the headgroups seems to be lower compared to the hydrophobic core. In pure POPC (Iwamoto and Oiki, 2013) or DOPC, the effect of the lipids on the KcsA channel limits opening. This limitation is overcome in clusters, where the channels can open cooperatively. The additional energy to enter the open state is provided by the fusion of the microdomains surrounding the KcsA channels, now sharing the energy cost by simultaneous opening.

Visscher et al. (Visscher et al., 2017) suggested a protein-protein interface between KcsA channels linked to cooperative opening. While this does not agree with our FRET measurements, the authors also suggested an additional unknown mechanism of interaction. A protein-protein interaction interface combined with the dependence on the physicochemical properties of the lipid membrane environment would consolidate both results.

In this work, we presented simultaneous single channel fluorescence and current data that allowed us to characterize in detail channel clustering and its effect on channel activity in real time. The results will likely find a widespread application on a variety of membrane proteins, in particular for proteins, for which oligomerization is an essential part of their physiological function. While ion channels are mainly described to cluster via protein-protein interactions (Kim et al., 1995; Tiffany et al., 2000; Dixon et al., 2012; Olesen et al., 2013; Dixon et al., 2015), high expression will lead to surface “crowding”, necessarily leading also to lipid-mediated ion channel interaction. For instance, Kaluin et al., (Kaulin et al., 1998), suggested that large conductance from antibiotic syringomycin E channels was a result of clustered channels gating cooperatively. Similarly, Grage et al. (Grage et al., 2011) proposed a coupled-gating mechanism for MscL channels, and, in the voltage-gated prokaryotic potassium channel KvAP, relatively high fluctuations in macroscopic currents are observed that exceeds normal “shot noise” (Ruta et al., 2005; Faure et al., 2012; Faure et al., 2014). This increased noise indicates a cooperative operation of the channels and possibly clustering. Clustering therefore seems essential not only for bacterial toxins or scaffolding proteins but may be a general property of many integral membrane proteins with significant impact on protein function.

## Data Availability

The raw data supporting the conclusions of this article will be made available by the authors, without undue reservation.

## Footnotes

1 Center Wavelength/Bandwidth.

2 Measured from Trp 87 to Trp 113.
